# CFD Modelling Validated by PIV of Hydrodynamics in a Raceway Bioreactor: Dead Zone Detection and Flow Field Analysis

**DOI:** 10.3390/bioengineering13030285

**Published:** 2026-02-28

**Authors:** Luis Alberto Zamora-Campos, Daniel Eduardo Rivera-Arreola, Rafael Rojas-Hernández, Valentín Trujillo-Mora, Marco Antonio Márquez-Vera, Julio César Salgado-Ramírez, Arturo Cadena-Ramírez

**Affiliations:** 1 Departamento de Posgrado, Universidad Politécnica de Pachuca, Zempoala 43830, Hidalgo, Mexico; alberto.zamora.campos@gmail.com (L.A.Z.-C.); eduardorivera@upp.edu.mx (D.E.R.-A.); marquez@upp.edu.mx (M.A.M.-V.); 2 Centro Universitario, Ingeniería en Computación, Universidad Autónoma del Estado de Mexico, Zumpango 55600, Estado de Mexico, Mexico; rrojashe@uaemex.mx (R.R.-H.); vtrujillom@uaemex.mx (V.T.-M.)

**Keywords:** raceway bioreactor, CFD model, abiotic medium, experimental validation

## Abstract

Raceway bioreactors are widely employed for microalgal production owing to their low construction and operational costs, in addition to their scalability benefits. Nonetheless, limited hydrodynamic studies are corroborated by computer models that have been experimentally validated. This paper delineates the methodology and validation of a computational fluid dynamics (CFD) model for a 10 L laboratory-scale Raceway bioreactor operating under abiotic conditions. In ANSYS Fluent, a multiphase technique was used with the RNG k–ε turbulence model, which is good for simulating flows that are curved or rotating in open-channels. Experimental validation was performed using Particle Image Velocimetry (PIV) at paddlewheel velocities of 20, 25, and 30 rpm. The CFD predictions showed a strong match with the experimental data, with a mean relative error of less than 8%. The examination of the flow field revealed the formation and subsequent reduction of low-velocity zones, depending on the intensity of agitation. Based on study on velocity distribution and Reynolds number, it was suggested that the design be changed so that the paddlewheel be moved to improve flow homogeneity without increasing energy use. The validated CFD model provides a reliable basis for improving the hydrodynamics, design, and operation of Raceway bioreactors. Additionally, it serves as a foundation for future research on biomass cultivation and expansion, facilitating the development of more efficient and sustainable microalgal production technologies.

## 1. Introduction

Raceway bioreactors are widely employed for microalgae cultivation because they offer low construction and operating costs and can be scaled by extending the channel loop while keeping a simple open-pond configuration [1]. They are used in both research and industrial settings for products and services that include biofuels, nutraceuticals, wastewater treatment, and CO_2_ capture [2]. A typical raceway consists of two open channels forming a closed circuit separated by a central baffle, and a paddlewheel drives circulation around the loop. The paddlewheel must supply enough mechanical energy to offset hydraulic losses, which are most pronounced at bends where pressure drops and velocity gradients intensify [1]. Because the culture is shallow and open to the atmosphere, the resulting flow field is spatially heterogeneous and sensitive to operating conditions. Poor circulation can generate low-velocity regions that reduce effective mixing and can increase the risk of sedimentation or uneven exposure to light and nutrients.

Hydrodynamics is therefore a central engineering variable in raceways. In open-channel systems, sufficiently turbulent circulation is often viewed as necessary to reduce microalgal sedimentation and to promote more uniform transport of biomass, nutrients, and light exposure [2,3]. Computational Fluid Dynamics (CFD) offers a practical route to analyze these coupled effects in realistic geometries, but its usefulness for design depends on rigorous validation against experiments [4]. To date, only a limited number of CFD studies on raceway ponds have incorporated direct experimental validation, including recent comparisons by Kumari et al. [5] and design-oriented models by Pandey and Premalatha [6], alongside broader bioreactor CFD developments such as Oliveira et al. [7] and real-scale raceway optimization work by Inostroza et al. [8]. These contributions support the promise of CFD, yet they also underscore that predicted velocity distributions can remain sensitive to turbulence closures and to how boundary conditions and free-surface effects are represented, which can limit reliability for design when assumptions are overly simplified [9]. In parallel, some studies have explored strategies to improve circulation and reduce energy demand, such as inclined or curved paddlewheels [10,11,12] and geometry and depth-dependent configurations that affect dead-zone formation and hydrodynamic efficiency [9,13]. Experimental characterization of paddlewheel-driven velocity fields, often using Particle Image Velocimetry (PIV), highlights the need to connect measured flow structures to modelling choices [14].

Despite the widespread use of open-channel raceways for large-scale microalgae cultivation, hydrodynamic modelling still shows an uneven balance between numerical sophistication and experimental corroboration. Planar, spatially resolved PIV measurements at laboratory scale have clarified how partitions and channel geometry shape velocity distributions [15,16]. In parallel, many CFD investigations emphasize performance proxies—such as light/dark cycling and particle tracking—for which biological inferences depend critically on the underlying hydrodynamics [17,18]. At larger scales, CFD has been used to optimize real raceway configurations and features such as bends and baffles, reinforcing the relevance of modelling for scale-up while highlighting the need for feasible validation strategies [8]. Recent studies on passive mixing concepts further illustrate the shift toward geometry-driven improvements that can be evaluated numerically when the flow model is trustworthy [19].

This study aims to enhance the comprehension and modeling of Raceway bioreactor hydrodynamics by constructing and testing a multiphase CFD model, supported by experimental PIV data. The practical objective is to obtain design-relevant hydrodynamic predictions at a computational cost compatible with iterative testing. Reynolds-averaged Navier–Stokes (RANS) modelling coupled to a two-equation k–ε closure is widely used for channel flows, and the RNG k–ε variant is often selected when curvature, swirl, and rotational effects are prominent [20,21]. Because predicted velocity fields can remain sensitive to modelling assumptions, quantitative validation against measurements is essential before CFD is used for design decisions [2,3]. For completeness, the full RANS–RNG k–ε formulation, model constants, and symbol definitions are provided in the Supplementary Materials (Supplementary S1; Equations (S1)–(S5) and Table S1). Details of the auxiliary definitions used in the implementation (e.g., eddy viscosity and strain-rate invariants) are reported in Supplementary S1 to keep the Introduction concise. Here, a multiphase CFD model of a 10 L raceway operated under abiotic conditions was constructed and tested with paddlewheel rotation at 20, 25, and 30 rpm [1]. Three linked hypotheses were tested: (i) the model reproduces PIV-derived, zone-averaged velocities across the loop with low relative error; (ii) increasing paddlewheel velocity reduces the extent of low-velocity dead zones; and (iii) a targeted change in paddlewheel position improves flow homogeneity without increasing operating velocity. The validated simulations match the experimental velocity data with a mean relative error below 8%, identify agitation-dependent low-velocity regions, and support a practical recommendation to relocate the paddlewheel to enhance circulation. These outcomes provide a validated hydrodynamic framework for improving raceway operation and for supporting future scale-up and cultivation studies. By linking validation to a concrete design variable, the study helps translate insight into actionable raceway improvements.

## 2. Materials and Methods

### 2.1. Bioreactor

**Figure 1:**
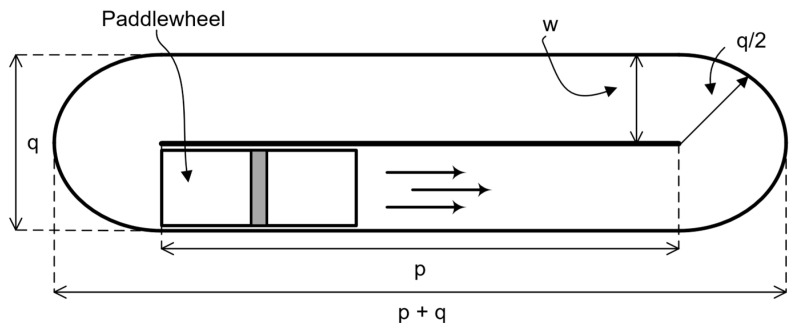
Schematic representation of the laboratory-scale raceway bioreactor employed in this study, showing the main geometric features used in both CFD simulations and experimental analysis. The geometric parameters were used to ensure consistency between numerical modelling and experimental PIV measurements.

The Raceway bioreactor utilized in this study has a total volume of 10 L. The length of the center baffle (p) is 0.50 m, the width of the channel (w) is 0.10 m, the total width (q) is 0.20 m, and the depth of the channel (h) is 0.08 m. Also, the system has a paddlewheel with a diameter of 0.17 m (Figure 1). The culture medium is abiotic and has a density (ρ) of 1000 kg·m^−3^ and a viscosity (μ) of 0.001 kg·m^−1^·s^−1^.

### 2.2. CFD Model

Computational Fluid Dynamics (CFD) simulations were performed using ANSYS Academic Teaching Mechanical-CFD 25 Task License© (Ansys Inc., Canonsburg, PA, USA) software, which applies the Finite Volume Method (FVM) for numerical discretization of the governing equations. A multiphase approach was employed to represent the interaction between the liquid phase (culture medium) and the surrounding gas phase (air) under open-channel conditions. The computational domain was discretized using a structured hexahedral mesh generated with the multizone approach, resulting in 89,622 nodes and 80,325 elements. This mesh resolution was selected to adequately represent the main geometric features of the raceway while maintaining numerical stability and reasonable computational cost. Turbulence was modelled using the two-equation RNG k–ε turbulence model. The complete transport equations for k and ε, together with the RNG-specific terms and auxiliary relations (eddy viscosity, strain-rate tensor, and related invariants), are reported in the Supplementary Materials (Supplementary S1, Equations (S1)–(S5)), and the corresponding nomenclature is summarized in Table S1. The model constants used were C_1_ε = 1.42, C_2_ε = 1.68, and C_3_ε = 0.0845. The multiphase model considers a homogeneous open-channel system with a prescribed free-surface height of 0.079 m (corresponds to the total depth of the channel). The air phase was defined as the primary phase, while the liquid phase was defined as the secondary phase. Gravitational acceleration was set to 9.81 m·s^−2^ acting in the vertical direction.

All reactor walls were assumed to be made of stainless steel and were modelled with a uniform roughness height of 1 μm. The raceway was treated as a closed-loop domain with no physical inlet or outlet. Flow circulation was not imposed through mass-flow boundary conditions; instead, a characteristic velocity consistent with paddlewheel operation was prescribed to represent the driving force of circulation. The paddlewheel itself was not explicitly resolved geometrically in the CFD domain; rather, its hydrodynamic effect was represented implicitly through the imposed flow conditions.

A turbulence intensity of 3% was specified for the characteristic velocities used in the simulations. These velocities were obtained by converting the paddlewheel rotational velocity into an equivalent linear velocity according to Equation (1).

(1) $$v=2\pi \frac{RPM}{60}\cdot r$$

For rotational velocities of 20, 25, and 30 rpm, the corresponding linear velocities were 0.178, 0.223, and 0.267 m·s^−1^, respectively, and these values were used as boundary conditions in the CFD model. The expression used to convert paddlewheel rotational velocity to the characteristic velocity employed in the analysis is also provided in Supplementary S1 (Equation (S6)). Steady-state simulations were conducted to evaluate the flow field under the different operating conditions. The objective was to determine whether the model adequately captured the dominant hydrodynamic features of the raceway, enabling its use for subsequent analysis of flow distribution, low-velocity regions, and potential design optimization and scale-up studies.

#### 2.2.1. Multiphase Approach

A homogeneous multiphase formulation was adopted to represent the abiotic water–air system under open-channel conditions. In this study, the free surface was assumed to remain stable, with no wave formation, interface deformation, or phase entrainment. Under these assumptions, both phases were treated as sharing a common velocity field, which is appropriate for analyzing time-average hydrodynamics relevant to mixing and circulation patterns in channeled open ponds [22].

More interface-resolving approaches, such as the Volume of Fluid (VOF) method, are typically required when transient free-surface dynamics or interfacial deformation are of interest. However, such effects were not the focus of the present work, which aimed to characterize average velocity fields rather than detailed surface phenomena [23].

#### 2.2.2. Computational Mesh Resolution

The computational mesh resolution was selected based on geometric fidelity, numerical stability, and convergence behavior. Mesh quality metrics, including skewness and orthogonality, were verified to fall within recommended limits for RANS simulations. Steady-state solutions exhibited stable residual convergence and consistent velocity fields across the computational domain [24,25]. A formal mesh-independence study was not conducted; therefore, the reported results should be interpreted as representative of the selected mesh resolution. Nevertheless, the good agreement observed between CFD predictions and experimental PIV measurements suggests that the adopted mesh is sufficient to capture the dominant hydrodynamic features relevant to the objectives of this study.

### 2.3. Experimental Data of the Raceway Bioreactor

The PIV data employed in this study originates from experiments previously conducted within the same research group and reported by Bautista-Monroy et al. [12,26,27]. These experimental datasets are under the custody of the corresponding authors of the present manuscript. Bautista-Monroy was a member of the research group involved in the experimental campaign, and the data was generated as part of a shared research effort.

**Figure 2:**
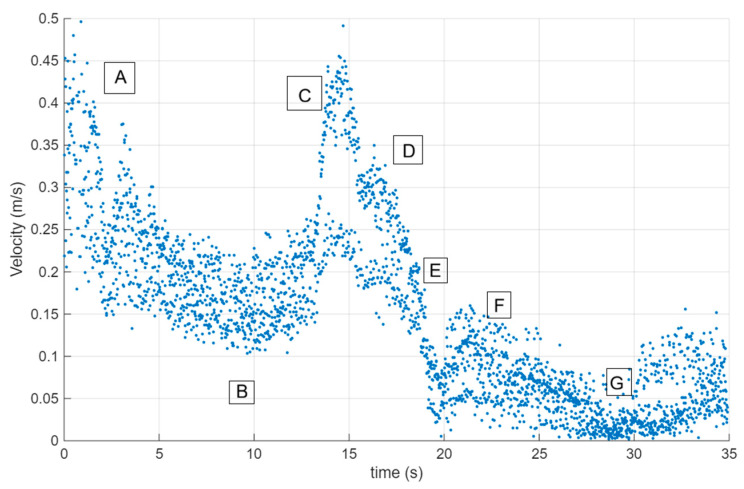
Example of instantaneous velocity field obtained from Particle Image Velocimetry (PIV) measurements in the raceway bioreactor using fluorescent tracer particles. The velocity vectors represent the in-plane flow distribution under paddlewheel-driven circulation and serve as the experimental reference for validating the CFD-predicted velocity fields. This data set captures both bulk circulation and localized velocity gradients characteristic of shallow open-channel raceways. The boxes with letters correspond to the areas of the raceway where the velocities were determined.

In the present work, the original experimental data were re-analyzed to extract velocity information consistent with the objectives of CFD validation. No original figures or raw images from the previous publication were reproduced. The experimental conditions adopted from the earlier study include the raceway geometry, paddlewheel rotational velocities (20, 25, and 30 rpm), fluid properties, and the definition of analysis zones. This approach ensures methodological consistency while enabling independent and complementary use of the experimental dataset for numerical model validation. Figure 2 shows an example of the velocity profile that was measured in the bioreactor using tracer particles.

**Figure 3:**
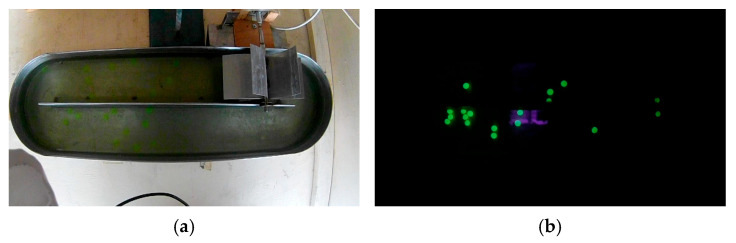
Experimental setup used for PIV data acquisition in the raceway bioreactor. (**a**) Top view of the bioreactor showing the camera positioning and illuminated measurement region used to capture planar velocity fields. (**b**) Hydrated hydrogel tracer particles containing fluorescein are employed to visualize the flow, selected for their size and density compatibility with the liquid phase to ensure accurate flow tracking.

Hydrated hydrogel microspheres containing fluorescein (diameter: 0.05 ± 0.01 mm; mean density: 0.654 kg·m^−3^) were used as tracer particles. Illumination was provided by an ultraviolet lamp (UVP, 6 W, wavelength 365 nm). A 12-megapixel video camera capable of recording at 120 frames per second was positioned 515 mm above the bioreactor (Figure 3). During the experiments, a paddlewheel with a diameter of 0.17 m was used to generate flow circulation, operating at rotational velocities of 20, 25, and 30 rpm.

**Figure 4:**
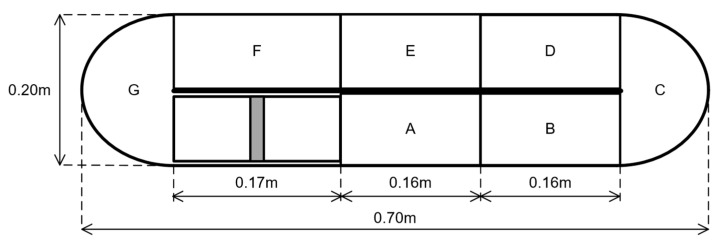
Definition of analysis zones within the raceway bioreactor used for zone-averaged velocity evaluation. The subdivision follows the methodology proposed by Bautista-Monroy et al., enabling quantitative comparison between CFD predictions and PIV measurements across hydrodynamically distinct regions. This zonal approach facilitates identification of low-velocity areas and assessment of spatial flow non-uniformity relevant to mixing performance.

The experimental velocity data were grouped into predefined zones of interest following the methodology proposed by Bautista-Monroy et al. [26], allowing the calculation of average flow velocities in different regions of the raceway. This zonal analysis facilitated a direct comparison between experimental measurements and CFD predictions (Figure 4).

The predefined zones of interest exhibit the following characteristics: Zone A is the inlet flow driven by the paddle wheel and is characterized by an average velocity like the initial conditions. Zone B is the flow along the straight channel before the first bend and is characterized by an average velocity like the initial conditions. Zone C is the flow in the first bend and is characterized by a slight decrease in average velocity compared to the initial conditions. Zone D is the flow after the first bend and is characterized by a second decrease in average velocity compared to Zone E. Zone E is the intermediate flow in the straight channel and is characterized by a third decrease in average velocity. Zone F is the flow along the straight channel before the second bend and is characterized by a fourth decrease in average flow velocity. Zone G is the flow in the second bend (outlet) and is characterized by the lowest average flow velocity along the entire path within the bioreactor.

## 3. Results

### 3.1. Raceway Bioreactor CFD

**Figure 5:**
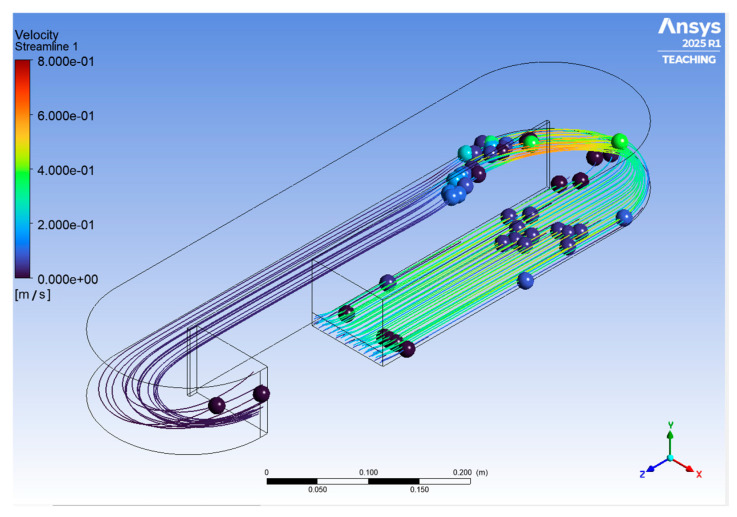
Spatial distribution of fluid velocity magnitude within the raceway bioreactor obtained from CFD simulations under paddlewheel-driven circulation.

The RNG k–ε turbulence model simulations were analyzed along the flow channel in the areas of interest that were set aside. We took the average velocities from these areas (Figure 5) and compared them to the experimental results (Figure 2). This method corresponds with Skiba’s assertion [28] that statistical averages are frequently employed to characterize physical quantities in fluid dynamics.

**Figure 6:**
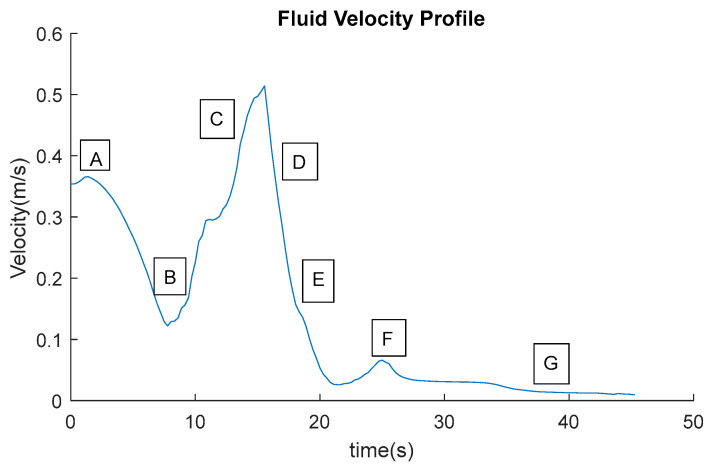
Fluid velocity profile in the raceway bioreactor at a paddlewheel angular velocity of 20 rpm, obtained from CFD simulations. The spatial distribution highlights the development of non-uniform flow conditions at low agitation intensity, with extended low-velocity regions forming downstream of the paddlewheel and near channel bends.

The bioreactor’s velocity profile makes it easy to see how the velocity changes in the channel volume. Figure 6 illustrates an example of a profile made with the new CFD model.

**Figure 7:**
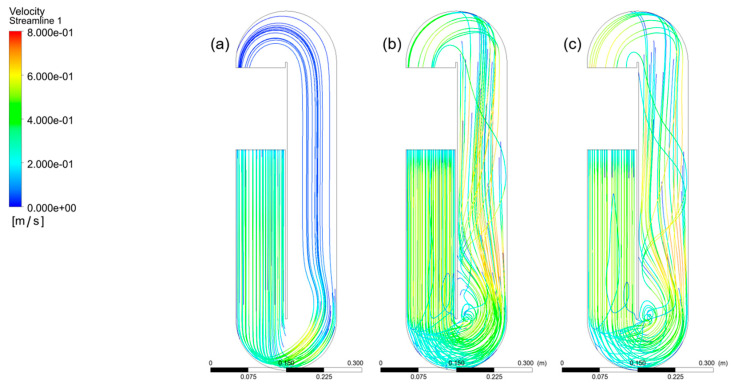
Comparison of CFD-predicted fluid velocity fields in the raceway bioreactor at paddlewheel rotational velocities of (**a**) 20 rpm, (**b**) 25 rpm, and (**c**) 30 rpm. The sequence illustrates the progressive enhancement of circulation intensity and velocity uniformity with increasing rotational velocity, as well as the reduction in the spatial extent of low-velocity regions.

Figure 7 shows the velocity fields that were found when the paddlewheel was turned at (a) 20, (b) 25, and (c) 30 rpm. The RNG k–ε turbulence model was used to simulate the whole flow route and reactor volume, which led to these results. For clarity, all symbols and derived quantities used for zone-averaged comparisons are defined in Supplementary S1 (Table S1).

**Figure 8:**
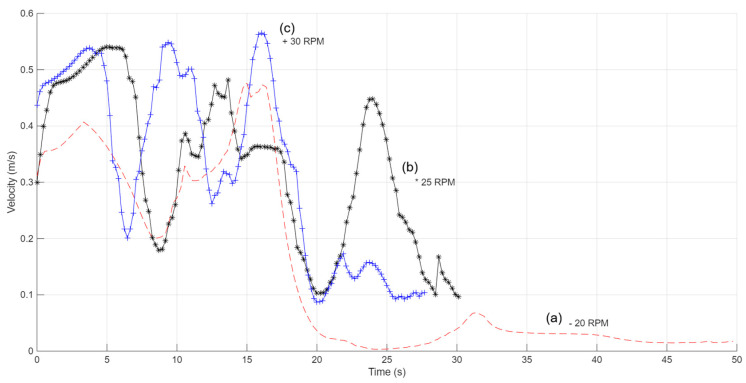
Spatial fluid velocity profiles in the raceway bioreactor obtained from CFD simulations at paddlewheel rotational velocities of (a) 20 rpm, (b) 25 rpm, and (c) 30 rpm. The profiles illustrate how increasing rotational velocity enhances circulation strength and reduces velocity heterogeneity across the channel.

At a paddlewheel angular velocity of 20 rpm (curve with the notation “-”), fluid velocities in regions A, B, and C vary from 0.2 to 0.5 m/s, which is what we want. On the other hand, zones D, E, F, and G have a velocity reduction below 0.1 m/s, which means that there are local pressure losses. When the paddlewheel turns at 25 rpm (curve with the notation “*”), the fluid velocities in regions A, B, C, D, and E range from 0.2 to 0.6 m/s. These velocities are fast enough to keep the turbulence needed for appropriate mixing in the culture medium. At the same time, the velocity in zones F and G drops to 0.1 m/s or lower. At 30 rpm (curve with the notation “+”), the velocities in areas A, B, C, D, E, and F stay between 0.2 and 0.6 m/s, which is the best range. In zone G of the bioreactor, the velocity drops, although values of around 0.1 m/s and a little higher are still seen (Figure 8).

The profiles show not only the velocity of the fluid but also the average time it takes for the tracer particles to make a full circuit through the bioreactor channels. Figure 8 shows that the particles finish a full cycle in about 50 s when the paddlewheel is moving at 20 rpm. When the paddlewheel is moving at 25 rpm, the average time goes down to about 32 s, and when it is moving at 30 rpm, it goes down to about 26 s. The average velocities and relative errors were calculated for each zone so that we could compare and check the flow velocities. Table 1 shows a summary of the velocities that were found.

After calculating the average fluid velocities in each of the predefined regions of interest using the experimental data (Table 1), the same procedure was followed with the average velocity data obtained through simulation. From this aggregated data, the overall relative error was calculated for each of the paddle wheel’s rotational speeds: 20, 25, and 30 RPM, respectively. This average relative error is summarized in Table 2. Note that for a speed of 20 RPM, the relative error is less than 8%; for a speed of 25 RPM, the relative error is less than 4%; and for a speed of 30 RPM, the relative error increases to 12%. However, the overall average relative error is less than 8%.

Dead zones, or regions with low fluid velocity and stagnation, can be identified within the flow field throughout the reactor volume. These zones are undesirable because they reduce the effective residence time and mix efficiency; hydrodynamically, they are inefficient regions that may lead to biomass sedimentation or local oxygen depletion.

As the paddlewheel rotational velocity increases, the extent of dead zones gradually decreases, as shown in Figure 7. Moreover, the streamlines in the Raceway bioreactor become more consistent and cohesive due to improved circulation and enhanced agitation. At a paddlewheel velocity of 30 rpm, vortex recirculation promotes better dispersion, resulting in a more uniform flow pattern.

To evaluate the flow regime within the culture medium, the Reynolds number was calculated (Equations (2) and (3)) using the average velocity data obtained from the designated regions of interest. This dimensionless parameter represents the ratio between inertial and viscous forces in the fluid. It provides insight into whether the flow regime is laminar or turbulent. In open-channel configurations, Reynolds numbers above 8000 indicate the onset of fully developed turbulent flow, according to Chisti [29]. The Reynolds numbers calculated for each bioreactor region under the different paddlewheel operating conditions are summarized in Table 3.

The Reynolds numbers were calculated using Equation (2).

(2) $$Re=\frac{\rho ud_{h}}{\mu }$$

where ρ is the density of the cultivation medium, u is the average flow velocity, dₕ is the hydraulic diameter of the channels, and μ is the dynamic viscosity of the medium.

The hydraulic diameter is defined by Equation (3):

(3) $$d_{h}=\;\frac{4wh}{w+2h}$$

where w is the channel width and h is the average depth of the cultivation fluid.

The Reynolds-number definition and the hydraulic-diameter expression adopted for the open-channel raceway are provided in Supplementary S1 (Equations (S1) and (S2); Table S1).

**Figure 9:**
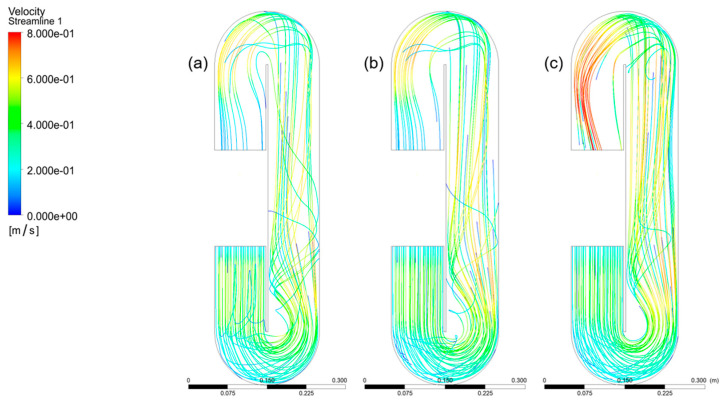
CFD-predicted fluid velocity fields in the raceway bioreactor at paddlewheel rotational velocities of (**a**) 20 rpm, (**b**) 25 rpm, and (**c**) 30 rpm, with the paddlewheel positioned at the center of the straight channel section. The velocity contours reveal the baseline circulation pattern and highlight the persistence of low-velocity regions downstream of the paddlewheel and near channel bends under this conventional placement.

This study shows that fluid velocity within the bioreactor can be significantly enhanced by relocating the paddlewheel. Improvements were observed when the paddlewheel was positioned either at the center of the straight channel or closer to the initial bend. Figure 9 illustrates the hydrodynamic response obtained when the agitator is placed at the center of the straight channel. The results are shown for paddlewheel rotational velocities of 20, 25, and 30 rpm.

**Figure 10:**
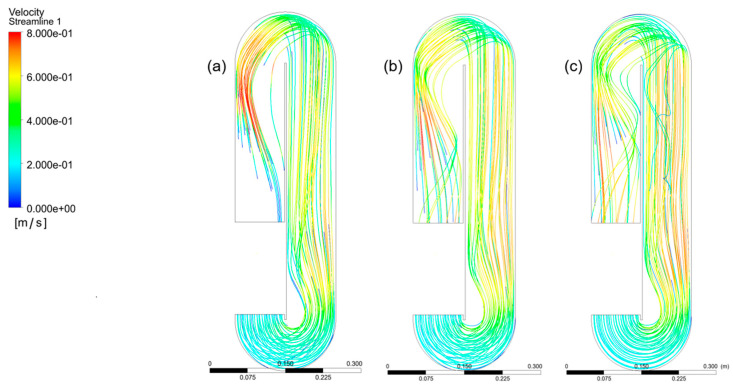
CFD-predicted fluid velocity fields in the raceway bioreactor at paddlewheel rotational velocities of (**a**) 20 rpm, (**b**) 25 rpm, and (**c**) 30 rpm, with the paddlewheel repositioned at the entrance of the first channel bend. Compared with the baseline configuration, this placement promotes a more uniform velocity distribution by mitigating flow separation and reducing the spatial extent of low-velocity regions.

Figure 10 shows a simulation illustrating the improvement achieved when the agitator is positioned at the beginning of the first bend, under paddlewheel angular velocities of 20, 25, and 30 rpm, respectively.

### 3.2. Validation Scope and Limitations

The validation between CFD predictions and PIV measurements was performed using zone-averaged velocity values, consistent with the spatial resolution and data processing methodology of the experimental study. As such, the comparison focuses on representative hydrodynamic magnitudes and flow patterns rather than pointwise correlations. Classical velocity correlation plots or coefficient-of-determination (R^2^) analyses require synchronized, point-by-point experimental and numerical datasets, which were not available for the present study. Similarly, experimental standard deviations could not be reported due to the absence of repeated measurements under identical conditions. These limitations are acknowledged; however, the obtained relative errors (3.9–12.1%) and the consistent agreement of spatial flow patterns support the suitability of the CFD model for diagnosing low-velocity regions and assessing design-related hydrodynamic trends.

## 4. Discussion

The validated CFD framework provides insights that extend beyond numerical agreement with experimental data and have direct implications for the design, scaling, and operation of raceway bioreactors. In the present work, the CFD–PIV agreement was within a mean relative error of <8%, with a maximum divergence of 12.1% at 30 rpm. This level of agreement is consistent with the range reported in prior raceway CFD studies that explicitly examined turbulence closure and paddlewheel-driven circulation patterns, including realizable k–ε RANS frameworks validated against PIV with deviations on the order of ~7% under comparable operational logic [6]. The above is promising for the proposed model since other works such as Kumari et al. [5] found differences of over 20% when they compared RANS and Large Eddy Simulation predictions to experimental data in Raceway ponds.

Similar numerical–experimental approaches have also been used to jointly interpret hydrodynamics and transport-relevant performance constraints in open raceway configurations, supporting the view that validated CFD can capture the dominant circulation structures needed for engineering decisions [2]. From a scalability perspective, the key value of a validated model lies in its ability to identify hydrodynamic patterns that persist under geometric similarity rather than in reproducing every local fluctuation. Real-scale and pilot-scale studies confirm that bends, baffle partitions, and channel curvature strongly condition velocity non-uniformity and the emergence of low-velocity regions, which then influence residence time and mixing quality [3]. Likewise, energy-oriented CFD design work highlights that a dominant component of losses is associated with sustaining circulation around 180° bends, and that geometric modifications (e.g., flow deflectors and baffle variants) can reduce pressure drops and dead zones while supporting more uniform velocities [4]. Together, these findings reinforce that scale-up should prioritize maintaining dynamically analogous regimes—particularly Reynolds-number ranges consistent with adequate suspension and circulation—rather than relying only on absolute velocity increases [2,5]. A recent dedicated review on raceway ponds further emphasizes that hydrodynamics remains one of the primary constraints shaping productivity and operational robustness at larger scale, motivating design rules that explicitly target mixing, shear, and dead-zone control [6].

Energy effectiveness emerges as a central theme when interpreting the CFD patterns. The simulations indicate that increasing paddlewheel velocity reduces the extent of low-velocity regions, but the broader literature shows that energy-efficient strategies frequently depend on how momentum is introduced and redistributed rather than on simply intensifying agitation. For example, studies combining CFD with experimental validation have shown that blade inclination and mixing-device design can improve mixing efficiency without proportionally increasing power demand, underscoring that geometric/kinematic tuning can be an energy-relevant lever [5]. Similarly, design-oriented CFD studies demonstrate that deflectors and bend-optimized configurations can lower hydraulic penalties at turns and reduce dead-zone severity, offering an alternative pathway to better mixing at constrained energy input [4]. At real scale, CFD-based optimization has even identified operating conditions that achieve adequate mixing at minimum energy consumption, strengthening the engineering case for design-first (rather than motor-first) optimization during scale-up [3].

The validated CFD model is most useful when treated as a diagnostic instrument for practical engineering. In operational terms, Reynolds-number maps and zone-resolved velocity patterns offer a direct way to localize hydrodynamically inefficient regions and to prioritize interventions where they can have the largest effect. Prior validated CFD analyses of raceways have explicitly examined how paddlewheel positioning, geometry ratios, and clearance influence flow distribution and vertical mixing indices, demonstrating that placement and configuration can be treated as design variables rather than fixed constraints [6]. In parallel, open-channel CFD studies integrating hydrodynamics with transport performance report that increasing characteristic circulation velocities can reduce settling propensity and modify near-surface uniformity, but the benefits may be spatially non-uniform, reinforcing the value of spatial diagnostics (rather than global averages) for engineering control [30]. These considerations support practical recommendations that focus on (i) mitigating bend-associated separation/slowdown, (ii) stabilizing circulation uniformity along the loop, and (iii) minimizing the footprint of persistent low-velocity pockets that can compromise mixing and suspension.

Beyond paddlewheel positioning, the broader field increasingly converges on geometry-driven or add-on interventions that can be assessed numerically once a flow model is credible. Passive devices intended to promote vertical mixing—such as vortex generators—have been studied to induce sustained secondary motion and improve vertical exchange downstream of bends, illustrating an emerging engineering direction for open raceways [19]. Complementary strategies to address dead zones have also been explored, including combining paddlewheel circulation with localized sparging or auxiliary injection to increase velocities in stagnant regions, which demonstrates that “targeted interventions” can be more efficient than uniform velocity increases [31]. Finally, an important motivation for strengthening hydrodynamics is that many contemporary raceway studies connect circulation and mixing patterns to light–dark cycling, cell trajectories, and even photosynthesis-informed productivity models; such couplings depend critically on the credibility of the underlying flow representation [17,18]. In this context, a validated hydrodynamic model provides not only a design tool for mixing uniformity, but also a defensible basis for future work that links hydrodynamics to growth or productivity indicators in a mechanistically consistent way.

In summary, the present CFD–PIV agreement (<8% mean relative error; 12.1% maximum divergence at 30 rpm) supports the model’s suitability for diagnosing flow non-uniformity and evaluating design-relevant trends in a raceway bioreactor. The discussion is therefore most appropriately framed around engineering implications: scale-up should focus on preserving dynamically analogous mixing regimes, energy effectiveness can be improved through design and placement rather than motor intensification, and practical interventions should prioritize bend-associated losses and persistent dead-zone regions. These conclusions align with recent validated CFD and scale-up studies in the field and reinforce the utility of CFD as a decision-support tool for energy-aware raceway design [6,13].

## 5. Conclusions

This study provides evidence of the reliability and predictive capability of the proposed CFD model for steady-state hydrodynamics in a 10 L raceway bioreactor. Validation against Particle Image Velocimetry (PIV) measurements resulted in an average relative error of less than 8%. This result demonstrates quantitative agreement between simulated and experimental velocity fields. The RNG k–ε turbulence model achieved a suitable balance between numerical accuracy and computational efficiency. It accurately reproduced flow behavior at paddlewheel rotational velocities of 20, 25, and 30 rpm. The simulations identified low-velocity regions primarily downstream of the paddlewheel and near channel bends. In these areas, the intensity of local turbulence decreased. These regions represent clear opportunities for hydrodynamic improvement through geometric modifications, such as paddlewheel repositioning. Apart from these localized zones, the calculated Reynolds numbers remained above the turbulent threshold. This indicates that fully developed turbulence was maintained under all evaluated operating conditions. Overall, the results demonstrate the availability of a validated and transferable CFD framework for the scale-up and design optimization of open-channel photobioreactors. This framework enables targeted strategies to enhance suspension stability and mixing uniformity while minimizing additional energy input.

## Figures and Tables

**Table 1:** Zone-averaged fluid velocities calculated for each predefined region of interest within the raceway bioreactor at the different paddlewheel rotational velocities.

Zone	20 RPM Velocity (m/s)	25 RPM Velocity (m/s)	30 RPM Velocity (m/s)
A	0.3195	0.3537	0.4053
B	0.3403	0.5274	0.5246
C	0.2867	0.3548	0.3867
D	0.0243	0.2508	0.3929
E	0.0326	0.2884	0.2662
F	0.0294	0.0985	0.1309
G	0.0172	0.0100	0.1039

**Table 2:** Summary of relative errors between simulated and experimental average velocities at all paddlewheel rotational velocities (20, 25, and 30 rpm).

Paddlewheel Velocity (RPM)	% Relative Error
20	7.6
25	3.9
30	12.1
Average % error	7.9

**Table 3:** Average Reynolds numbers calculated across the regions of interest under paddlewheel rotational velocities of 20, 25, and 30 RPM.

Zona	20 RPM	25 RPM	30 RPM
A	39,323	43,532	49,883
B	41,883	64,910	64,566
C	35,286	43,667	47,593
D	2990	30,867	48,356
E	4012	35,495	32,763
F	3618	12,123	16,110
G	2116	1230	12,787

## Data Availability

The data presented in this study are available upon request from the corresponding author because the hydrodynamic study is progressing to a second stage.

## Supplementary Materials

The following supporting information can be downloaded at: https://www.mdpi.com/article/10.3390/bioengineering13030285/s1, Full RANS–RNG k–ε equations and auxiliary relations (Equations (S1)–(S6)) and nomenclature table (Table S1).

## Abbreviations

The following abbreviations are used in this manuscript:

CFD	Computational Fluid Dynamics
fps	frames per second
FVM	Finite Volume Method
PIV	Particle Image Velocimetry
RANS	Reynolds-Averaged Navier–Stokes
RNG	Re-Normalization Group
RPM	Revolutions Per Minute
