# Supplementary material for: CFD Modelling Validated by PIV of Hydrodynamics in a Raceway Bioreactor: Dead Zone Detection and Flow Field Analysis
Source: Bioengineering (Basel). 2026 Feb 28;13(3):285. doi: 10.3390/bioengineering13030285 (PMC13024567; doi:10.3390/bioengineering13030285)
Supplement: Supplementary file 1 [file bioengineering-13-00285-s001.zip › bioengineering-3987670-supplementary.pdf]

Supplementary Materials S1

Turbulence-model equations and nomenclature used for the RNG k-ε simulations

S1.1. RNG k-ε transport equations (as used in the manuscript)

This Supplementary section aids to improve readability of the main manuscript while retaining full mathematical transparency. Equations (S1)–(S2) reproduce the RNG k-ε transport equations used in the study.

The Reynolds-average continuity equation is:

$$\frac{\partial}{\partial t}(\rho k) + \frac{\partial}{\partial x_i}(\rho k u_i) = \frac{\partial}{\partial x_j}\left(\alpha_k \mu_{eff} \frac{\partial k}{\partial x_j}\right) + G_k + G_b - \rho\varepsilon - Y_M + S_k \quad \ldots\ldots\ldots\ldots\text{.. (S1)}$$

$$\frac{\partial}{\partial t}(\rho \varepsilon) + \frac{\partial}{\partial x_i}(\rho \varepsilon u_i) = \frac{\partial}{\partial x_j}\left(\alpha_\varepsilon \mu_{eff} \frac{\partial \varepsilon}{\partial x_j}\right) + C_{1\varepsilon}\frac{\varepsilon}{k}(G_k + G_{3\varepsilon}G_b) - C_{2\varepsilon}\rho\frac{\varepsilon^2}{k} - R_\varepsilon + S_\varepsilon \quad \ldots\ldots \text{(S2)}$$

Where:

- ρ: density of the fluid phase of interest (kg m^{-3}).
- u_i: i-th component of mean velocity (m s^{-1}).
- x_i, x_j: Cartesian coordinates (m).
- t: time (s).
- k: turbulent kinetic energy (m^2 s^{-2}).
- ε: turbulent dissipation rate (m^2 s^{-3}).
- μ: dynamic viscosity (Pa s).
- μ_t: turbulent (eddy) viscosity (Pa s).
- μ_{eff}: effective viscosity, $\mu_{eff} = \mu + \mu_t$ (Pa s).
- α_k, α_ε: inverse effective turbulent Prandtl numbers for k and ε, respectively (dimensionless).
- G_k: production of k due to mean velocity gradients (W m^{-3}).
- G_b: production of k due to buoyancy (W m^{-3}).
- Y_M: contribution of fluctuating dilatation in compressible turbulence to the dissipation rate (W m^{-3}).
- S_k, S_ε: user-defined source terms for k and ε (W m^{-3}).
- $C_{1\varepsilon}$, $C_{2\varepsilon}$, $C_{3\varepsilon}$: model coefficients (dimensionless).
- R_ε: RNG additional term in the ε equation (W m^{-3}), defined in Section S1.2.

S1.2. RNG-specific terms, viscosity closure, and constants

The RNG k-ε model differs from the standard k-ε model primarily through the additional term R_ε in the ε-transport equation, analytically derived turbulent Prandtl numbers, and RNG-derived constants. Equations (S3)–(S5) provide the auxiliary definitions required to interpret Equation (S2) unambiguously.

$$\mu_t = \rho C_\mu \frac{k^2}{\varepsilon} \quad \ldots\ldots\ldots\ldots \text{(S3)}$$

$$R_\varepsilon = \frac{\rho C_\mu \eta^3 (1 - \frac{\eta}{\eta_0})}{(1+\beta\eta^3)} \frac{\varepsilon^2}{k} \quad \ldots\ldots\ldots\ldots \text{(S4)}$$

$$\eta \equiv S\frac{k}{\varepsilon},\ with\ S \equiv \sqrt{2S_{ij}S_{ij}},\ S_{ij} \equiv \frac{1}{2}\left(\frac{\partial u_i}{\partial x_j} + \frac{\partial u_j}{\partial x_i}\right) \text{………… (S5)}$$

Model constants used for the RNG k-ε closure (ANSYS Fluent implementation) include C_μ = 0.0845, η_0 = 4.38, and β = 0.012. In the high-Reynolds-number limit, $\alpha_k \approx \alpha_\varepsilon \approx$ 1.393. The manuscript uses $C_{1\varepsilon}$ = 1.42 and $C_{2\varepsilon}$ = 1.68 in Equation (S2). The coefficient $C_{3\varepsilon}$ is the buoyancy coefficient in the ε equation and is commonly treated as stability-dependent (0–1), consistent with the description in the main manuscript.

S1.3. Derived quantities used for boundary conditions and flow-regime assessment

Paddlewheel tip speed (linear velocity from rotational speed). To convert paddlewheel rotation rate N (rpm) to a characteristic linear speed *U* (m·s−1), the circumferential speed based on paddlewheel diameter *D* (m) is used:

$$U = \frac{\pi D N}{60} \text{……… (S6)}$$

This relation yields the values reported in the manuscript for *D* = 0.17 m and *N* = 20, 25, and 30 rpm.

Table. S1. Nomenclature

Symbol	Definition	Unit
R_ε	RNG additional term in ε equation	W m^{-3}
C_μ	RNG viscosity coefficient	–
$C_{1\varepsilon}$	Production coefficient in ε equation	–
$C_{2\varepsilon}$	Dissipation coefficient in ε equation	–
$C_{3\varepsilon}$	Buoyancy coefficient in ε equation	–
η	RNG strain parameter (S k/ε)	–
η_0	RNG constant (4.38)	–
β	RNG constant (0.012)	–
S	Mean strain-rate magnitude	s^{-1}
S_{ij}	Mean strain-rate tensor	s^{-1}

References for Supplementary S1

1. Yakhot, V.; Orszag, S.; Thangam, S.; Gatski, T.; Speziale, C. G. Development of turbulence models for shear flows by a double expansion technique. *Physics of Fluids A* 1992, 4(7), 1510–1520. https://doi.org/10.1063/1.858424
2. Fluent Inc. Standard, RNG, and Realizable k-ε Models Theory. 2006 (documentation extract). Available at: https://courses.washington.edu/mengr544/handouts-10/Fluent-k-epsilon.pdf
3. Yakhot, V.; Orszag, S. A. Renormalization group analysis of turbulence. I. Basic theory. *Journal of Scientific Computing* 1986, 1, 3–51. https://doi.org/10.1007/BF01061452
